# Targeted Therapy; From Advanced Melanoma to the Adjuvant Setting

**DOI:** 10.3389/fonc.2013.00205

**Published:** 2013-08-09

**Authors:** Antonio Ahn, Michael R. Eccles

**Affiliations:** ^1^Developmental Genetics and Pathology Laboratory, Department of Pathology, Dunedin School of Medicine, University of OtagoDunedin, New Zealand

Targeted therapy has revolutionized treatment for advanced melanoma. Clinical trials have demonstrated unprecedented survival benefits in advanced melanoma patients treated with Vemurafenib (1, 2). Vemurafenib is a targeted inhibitor that specifically binds to mutant BRAF proteins containing V600E or V600K amino acid substitutions, preventing constitutive activation of the mitogen-activated protein kinase (MAPK) pathway, and resulting in antitumor effects of inhibition of cell proliferation and apoptosis induction (3).

New treatments are evolving rapidly in this area. The FDA has approved two other monotherapeutic drugs, Dabrafenib and Trametinib, which are also inhibitors of growth stimulatory effects of mutant BRAF, or its downstream effector pathways, and these have proven to improve survival rates (4, 5). Moreover, clinical trials are demonstrating further prolonged survival in addition to reduced treatment related toxic side-effects through combinatorial use of several of these targeted drugs (6). However, there is a great downside to targeted therapy in advanced melanoma: in practically all cases, drug resistance inevitably develops, and patient death inexorably follows (2, 6).

Stage IIB-C and stage III melanomas have a lower disease burden than stage IV melanoma, and yet these melanomas are at a significant risk of tumor recurrence following surgical resection (7). Currently there is a high demand for new and effective adjuvant treatments to mitigate the risk of recurrence, and there are a number of adjuvant therapies under investigation for stage IIB-C and stage III patients. The only FDA-approved adjuvant drugs for melanoma are interferon-alpha and pegylated interferon, which marginally improve overall survival (OS) for high-risk recurrent tumors (8). With the progress of targeted therapies in advanced melanoma and the need for better adjuvant drugs, many are now asking whether precision treatment could be used at an earlier stage of melanoma diagnosis in the adjuvant setting, which accounts for the majority of melanoma diagnoses. Indeed, a number of adjuvant clinical trials using targeted therapies for the treatment of stage IIC and stage III melanomas have now been initiated.

Several targeted monotherapies and combination therapies are currently being evaluated for melanoma treatment in the adjuvant setting in both stage IIC and stage III melanomas (clinical trials NCT-01667419, NCT01682083, NCT00553618, NCT01782508, and NCT01682213). However, because the risk of recurrence is less than 100% for these patients, multiple patients would need to be treated for every one patient who would receive benefit from the adjuvant therapy (9). Prognostic biomarkers are therefore needed to predict melanoma recurrence, but to date good prognostic biomarkers that accurately predict the outcome of stage IIB-C or stage III melanomas are lacking.

Prognostic markers determine the risk of tumor recurrence as a result of growth of cancerous cells that have escaped surgical resection, most likely due to metastasis, and as such these cancer cells are undetectable at the time of diagnosis. Indeed, initial presentation of recurrence was local in 10.9%, in transit in 9.9%, involving a regional lymph node in 34.4%, and at a distant site in 44.9% of patients with metastasis (9). Several studies have demonstrated the presence of *BRAF* mutations as a marker of poor prognosis in both the metastatic and locally advanced settings. This is important because targeted therapy could help to eliminate metastasized cells that harbor the *BRAF* mutation.

*BRAF* mutation predicts shorter OS in stage IV melanoma (10), which is consistent with clinical outcomes of tumor regression upon Vemurafenib administration in advanced melanoma patients; inhibition of a marker that is directly associated with poor prognosis results in prolonged survival. In stage III resected tumors *BRAF* mutations are associated with a significantly shorter DFS and OS (11, 12), and they have been shown to promote metastasis through mechanistic studies, albeit presumably associated with the progression and growth of the metastatic disease rather than the initiation of metastasis (13). Thus, for *BRAF* mutation-positive stage III patients, adjuvant targeted therapy may be of benefit. In contrast, *BRAF* mutation does not appear to have significant impact on prognosis in stage I or stage II melanomas. Numerous studies have shown the *BRAF* mutation does not affect the Disease Free Interval (DFI) or OS after surgical resection of melanomas at these stages (14–16), and thus it does not influence tumor recurrence.

In deciding whether to use a targeted treatment for melanoma in the adjuvant setting, either for stage III or stage IIB-C, it is important to consider whether the tumor cells that avoid surgical resection, presumably due to early metastasis, would continue to harbor the mutation being targeted (e.g., *BRAF*). This can be guided by the observations that most primary melanomas with a *BRAF* mutation have paired secondary lesions also harboring the mutation (17). This may be explained by the fact that *BRAF* mutations are acquired during the early stages of tumor progression, for example during radial to vertical growth phase (18), resulting in a larger portion of the primary tumor with the mutation. Clones that acquire metastatic capability will most likely therefore possess the *BRAF* mutation and so *BRAF* mutant targeted therapy should work.

As *BRAF* mutations influence tumor growth, it is unlikely that they would confer metastatic capability. For instance, primary tumors with a *BRAF* V600E mutation may frequently be paired with secondary lesions without the mutation (17, 19). Colombino and colleagues found 6 of 44 *BRAF* mutant primary melanoma patients whose primary melanomas were positive for the *BRAF* V600E mutation, yet had a *BRAF* wild-type secondary lesion (17). To explain this, Yancovitz and colleagues showed intra-tumoral heterogeneity of clones with respect to *BRAF* V600E mutation status, and concluded *BRAF* mutations were not necessary for metastasis (19). Importantly, administration of targeted therapy inhibiting mutant BRAF to *BRAF* wild-type patients has been shown not only to have absence of benefit, but can also cause a growth advantage in those tumor cells by paradoxically stimulating the MAPK pathway (20).

In addition, *BRAF* wild-type primary tumors may be paired with mutant *BRAF* secondary tumors, due to the acquisition of the *BRAF* mutation at the secondary site. These patients would be likely to benefit from adjuvant targeted therapy, with the degree of benefit depending on how early the *BRAF* mutation had occurred during the cellular evolution of the secondary tumor. Mutant *BRAF* might therefore be a useful therapeutic target in the metastatic lesions of stage III melanoma patients for this reason, as compared to those patients with a localized melanoma of stage IIB-C. Mann et al. (12) for example, have shown that *BRAF* mutation status may also be combined with an expression signature to enhance the ability to predict melanoma recurrence.

Although it might be questioned whether patients with stage IIB melanoma should also be included in the adjuvant therapy clinical trials, despite the promising leads mentioned above there is currently not a lot of prognostic information that supports the use of mutant *BRAF* targeted therapy to treat stage III melanomas in the adjuvant setting, and even less information to support the use of *BRAF* targeted therapy to treat stage IIB-C melanomas. While randomized phase III clinical trials are currently recruiting to evaluate the use of *BRAF* targeted therapy for stage IIC and III melanomas in the adjuvant setting as an alternative to interferon drugs, these treatments are not without the potential to develop some or all of the adverse side-effects of the *BRAF* targeted therapies (2). In addition, *BRAF* mutations activate the MAPK pathway, which is associated with increased MITF expression (21). Therefore inhibiting *BRAF* activity in stage II melanomas could lead to repression of both MITF and miR-211 expression in those tumors (see He et al., submitted), and if the environmental signals are conducive, then this could subsequently cause up-regulated expression of BRN2, a factor that is thought to be associated with phenotype switching (22), and so induce metastasis.

Based on the points we have outlined above, it is our opinion that treatment of stage II melanoma with BRAF inhibitors in the absence of data from suitable prognostic biomarkers that adequately predict the outcome of stage II melanomas, could lead to adverse outcomes for these patients. It is hoped that these trials will provide insight as to how targeted therapies perform in the adjuvant setting in patients with tumor recurrence. The identification of which patients have a high-risk of recurrence of melanoma requires better biomarkers of tumor progression and prognosis. In addition, new biomarkers of melanoma metastasis are needed, together with the concurrent development of new or existing drugs for use in the adjuvant setting.
